# The error of estimated GFR in predialysis care

**DOI:** 10.1038/s41598-024-55022-8

**Published:** 2024-03-03

**Authors:** Beatriz Escamilla-Cabrera, Sergio Luis-Lima, Eduardo Gallego-Valcarce, Nuria Victoria Sánchez-Dorta, Natalia Negrín-Mena, Laura Díaz-Martín, Coriolano Cruz-Perera, Ana Monserrat Hernández-Valles, Federico González-Rinne, María José Rodríguez-Gamboa, Sara Estupiñán-Torres, Rosa Miquel-Rodríguez, María Ángeles Cobo-Caso, Patricia Delgado-Mallén, Gema Fernández-Suárez, Ana González-Rinne, Grimanesa Hernández-Barroso, Alejandra González-Delgado, Armando Torres-Ramírez, Alejandro Jiménez-Sosa, Alberto Ortiz, Flavio Gaspari, Domingo Hernández-Marrero, Esteban Luis Porrini

**Affiliations:** 1 Nephrology Department, Complejo Hospitalario Universitario de Canarias, La Laguna, Spain; 2 Department of Laboratory Medicine, Complejo Hospitalario Universitario de Canarias, Tenerife, Spain; 3 Nephrology Department, Hospital Universitario Fundación Alcorcón, Madrid, Spain; 4 Laboratory of Renal Function (LFR), Faculty of Medicine, Complejo Hospitalario Universitario de Canarias, University of La Laguna, La Laguna, Spain; 5 Research Unit, Hospital Universitario de Canarias, La Laguna, Spain; 6 Faculty of Medicine, Universidad Autónoma de Madrid. IIS-Fundación Jiménez Díaz. RICORS, Madrid, Spain; 7 Instituto de Tecnologías Biomédicas (ITB), Faculty of Medicine, University of La Laguna, La Laguna, Spain; 8 Facultad de Medicina, Universidad de La Laguna, La Laguna, Spain

**Keywords:** Nephrology, Kidney diseases, Renal replacement therapy

## Abstract

The error of estimated glomerular filtration rate (eGFR) and its consequences in predialysis are unknown. In this prospective multicentre study, 315 predialysis patients underwent measured GFR (mGFR) by the clearance of iohexol and eGFR by 52 formulas. Agreement between eGFR and mGFR was evaluated by concordance correlation coefficient (CCC), total deviation index (TDI) and coverage probability (CP). In a sub-analysis we assessed the impact of eGFR error on decision-making as (i) initiating dialysis, (ii) preparation for renal replacement therapy (RRT) and (iii) continuing clinical follow-up. For this sub-analysis, patients who started RRT due to clinical indications (uremia, fluid overload, etc.) were excluded. eGFR had scarce precision and accuracy in reflecting mGFR (average CCC 0.6, TDI 70% and *cp* 22%) both in creatinine- and cystatin-based formulas. Variations -larger than 10 ml/min- between mGFR and eGFR were frequent. The error of formulas would have suggested (a) premature preparation for RTT in 14% of stable patients evaluated by mGFR; (b) to continue clinical follow-up in 59% of subjects with indication for RTT preparation due to low GFRm and (c) to delay dialysis in all asymptomatic patients (n = 6) in whom RRT was indicated based on very low mGFR. The error of formulas in predialysis was frequent and large and may have consequences in clinical care.

## Introduction

Major clinical objectives in pre-dialysis care include delaying the progression of chronic kidney disease (CKD), treating and preventing complications related with renal disease, preparing patients for renal replacement therapy (RRT) and deciding the time to start dialysis. The achievement of these objectives is associated with better clinical outcomes^1,2^.

Diverse scientific societies of Nephrology proposed guidelines to standardize clinical care for patients in pre-dyalisis^3–9^. In general, pre-dialysis care must start when GFR is below 30 ml/min 1.73 m^2^^3^, the evaluation of living donor transplantation with GFR below 20 ml/min 1.73 m^2^^4^ and preparation for RTT i.e. the creation of vascular access GFR is less than 15 ml/min/1.73 m^2^^5–7^. All guidelines agree on the recommendation to start RRT in the presence of uraemic symptoms or volume overload, independently of the level of GFR^3–9^. However, in the absence of symptomatology, the recommendations of guidelines are quite heterogeneous. KDIGO guidelines suggest initiation of dialysis in symptomatic patients, which usually -but not always- occurs with GFR between 5–10 ml/min/1.73 m^2^^4^. The Spanish guidelines recommend to start RRT with GFR levels below 10 mL/min/1.73 m^2^ or higher if there are other factors which recommend an earlier starting^5,6^. Others guidelines suggest to start RRT with GFR around 5–7 ml/min/1.73 m^2^^7–9^ or 8–10 ml/min/1.73 m^2^^9^. All these information together makes crucial the use of a reliable evaluation of GFR in pre-dialysis care^7,10^.

In clinical practice, renal function is estimated by formulas or 24 h creatinine clearance^11^. However, the reliability of these methods has been questioned^12–17^. The average error of estimated GFR (eGFR) is about ± 30% of measured GFR (mGFR) and in 10–20% of the cases this error is even larger^14,15^. This variability may be relevant in predialysis. For example, in a patient with mGFR of 18 ml/min, eGFR may theoretically range from 12 to 24 ml/min. In another with mGFR of 10 ml/min, eGFR may vary from 6 to 15 ml/min^15^*.* Thus, the initiation or the preparation for dialysis may be anticipated or delayed following under- or overestimation of real renal function^14^. However, the magnitude of this error and its clinical impact on decision making in pre-dialysis care have not been evaluated.

We analyzed the error of a large group of formulas and its possible consequences in decision making in patients in predialysis who underwent measured GFR with a gold standard procedure, the plasma clearance of iohexol.

## Material and methods

### Study design

This is a prospective study that involves two Spanish centers (Hospital Universitario de Canarias -HUC- and Hospital Universitario Fundación de Alcorcón -HUFA). According to protocol, at baseline, all patients underwent the plasma clearance of iohexol and were followed to evaluate diverse outcomes (a) the error between measured GFR—(mGFR) and estimated GFR (eGFR) by diverse formulas; (b) the impact of this error in decision making and (c) the consequences of the error of eGFR in major outcomes in predialysis care. The present study shows the analysis of the first two outcomes. Thus, the results of the present study include the agreement between eGFR by diverse formulas and mGFR and the clinical decisions taken with mGFR and those that would have been taken had we used eGFR instead of mGFR in the immediate follow-up. The first patient was included in july 2016 and the last in January 2020. The patients of the HUFA are a representative group of subjects of the predialysis outpatient clinic in that centre. In both centres, patients with advanced CKD are included in predialysis care when they reach GFR levels < 30 ml/min. The protocol is in line with the Declaration of Helsinki and was approved by the Ethic Committee of the HUC.

### Patients

Inclusion criteria: age > 18 years; GFR < 30 ml/min (MDRD), clinical stability i.e. absence of uremic symptoms, fluid overload or electrolytic disorders that require urgent dialysis or acute infections or cardiovascular events; capacity to understand the protocol. Exclusion criteria: candidate for conservative care in whom dialysis is not indicated, i.e. short life expectancy, uncontrolled neoplasms, terminal heart disease; severe psychiatric or cognitive disorders. All patients provided written informed consent to participate.

### Patient care

It was carried out following current guidelines^3,4,10,11^, including regular clinical evaluation, assessment and treatment of the cause of renal disease and diverse co-morbidities. The latter include diabetes, hypertension, dyslipidemia, hyperuricemia and cardiovascular disease i.e.valvular heart disease, heart failure, ischaemic heart disease, peripheral vascular disease, stroke and specific treatment for these diseases. Specific treatment for end-stage CKD, such as anaemia drugs for CKD-MBD, etc., was followed by guidelines^4,15^ In each visit, patients were asked about uremic signs and symptoms, such as fatigue, nausea, vomiting, pruritus or encephalopathy. The information was collected from the patient's medical records and stored in an on-line database created ad hoc using the REDCap facility of the University of Vanderbilt (https://projectredcap.org/).

Blood and urine tests included serum creatinine, 24 h creatinine and urea clearances, proteinuria; electrolytes and acid–base, calcium, phosphorus, parathyroid hormone and vitamin-D; hemogram, iron metabolism and lipid profile.

### Measured GFR by the plasma clearance of iohexol

Five mL of iohexol (Omnipaque 300, GE-Healthcare) were injected intravenously for 2 min. Afterwards, venous or capillary blood (obtained by finger prick) samples were taken at 120, 180, 240, 300, 360, 420 and 480 min. This is the protocol used in the laboratory for cases with expected GFR below 30 ml/min, as described by Gaspari et al.^18^. This “long” procedure has good agreement with other methods and with more complete pharmacokinetic analyses^19^. Iohexol was measured in plasma or dried blood spot (DBS)^20^. Both methods (plasma or DBS) are interchangeable^20^. Iohexol was measured by HPLC–UV at the Laboratory of Renal Function of the ULL (http://lfr.ecihucan.es/). The clearance of iohexol was calculated according to a 1compartment model (CL1) by the formula: CL1 = Dose/ AUC, where AUC is the area under the plasma concentration time curve from time equal zero to infinity. The plasma clearances were then corrected using the Bröchner-Mortensen equation^21^.

### Estimated GFR

Simultaneously to the clearance of iohexol, serum creatinine and cystatin-c were determined to calculate eGFR by 52 formulas. The algorithms selected and their references are shown in the web page http://lfr.ecihucan.es/. The agreement between eGFR and mGFR was evaluated with the formulas unadjusted for body surface area (BSA). The adjustment by BSA is based on premises no longer sustained today by scientific support. In brief, the idea of adjusting GFR for BSA is derived from the hypothesis (from the nineteenth century) that metabolic rate was proportional to BSA^22^. This assumption probed to the incorrect: BSA has no clear correlation with metabolic demand or GFR^23,24^. By the other hand, GFR is not necessarily correlated with BSA or the number of glomeruli^25,26^. Also, adjusting for BSA artificially reduces GFR in obese subjects and eliminated gender differences in renal function. In this line, several critiques to the adjustment have been raised^27–29^.When eGFR was already adjusted, we reversed the adjustment of the result by applying the following formula (GFR unadjusted = GFR adjusted × BSA/1.73). BSA was calculated by the Du-Bois and Du-Bois formula^30^. Finally, to promote comparability with other studies we tested the agreement between eGFR and mGFR adjusted by BSA.

Serum creatinine was measured by an Isotope Dilution Mass Spectrometry-traceable creatinine assay (Cobas c711 module, Roche) and cystatin C levels by immunonephelometry (BN II System, Siemens Healthcare), calibrated with ERM-DA471/IFCC.

### Outcomes

#### Agreement between eGFR and mGFR

This aspect was evaluated by specific statistics of agreement for continuous variables, as proposed previously. Stadistics used to assess the agreement between mGFR and eGFR should consider precision and accuracy, and must provide confidence intervals. This is provided by the concordance correlation coefficient (CCC), total deviation index (TDI) and coverage probability(cp)^14,31^. CCC varies from 0 to 1 and combines components of accuracy and precision. A CCC > 0.90 reflects optimal concordance between measurements. TDI captures a large proportion of data within a boundary for allowed differences between measurements. Empirical TDI was calculated for a theoretical TDI of 10% and a cp of 90%. We defined a priori that the acceptable bias between eGFR and mGFR should be at least 10%. This is based on the reproducibility of the method in our laboratory: < 7%. Cp varies from 0 to 1 and estimates whether a given TDI is less than a pre-specified fixed percentage. We used these methods since other tests like correlation, mean and mean percent errors, Student’s *t*- test, linear regression, quadratic mean, and the percentage of estimations falling within defined margins of error are not suitable for agreement analysis. Pearson’s correlation coefficient does not take into account any differences in values between the groups being compared. Mean and mean percentage errors represent the mean of the difference between eGFR and mGFR for each patient. However large differences between mGFR and eGFR can be cancelled by equal numerical values.

Also, we analysed the error of eGFR in different subgroups such diabetes and elderly patients (over 65 years). We understand the complexity of the statistics of agreement, so, to help the reader, we selected examples of cases to provide a clear idea of the variability of eGFR. These examples must be evaluated in the general context of the agreement tests explained above. To promote comparability between studies ,we performed the p30 and p10 for each formula.

### Sensitivity analysis

#### Impact of the error of eGFR in clinical decisions

After the assessment of mGFR, three major clinical decisions were taken in the immediate follow-up.

(A) *To continue with clinical management* in stable patients without uremic symptoms or untreatable fluid overload, in whom mGFR is > 15 ml/min.
(B) *To start the preparation for RRT* in patients with progressive decline of GFR reaching values between 10–15 ml/min and/or worsening of their previous clinical status; to decide dialysis modality, create vascular access, place peritoneal dialysis catheter or evaluate for pre-emptive living donor transplantation.
(C) *To initiate of RRT in symptomatic patients, irrespective of the level of mGFR*: in patients with uremic symptoms: pruritus, cognitive impairment, asterixis, nausea or anorexia, deterioration in nutritional status, serositis; volume overload; severe electrolyte disturbances.
(D) *To initiate of RRT with very low mGFR* < *10 ml/min but without symptoms of uremia or volume overload.*

For the sensitivity analysis those patients that initiated dialysis based only on clinical symptoms –fluid overload, uremia-(group-C: n = 18) were excluded since GFR values were not considered for the initiation of dialysis. Finally, we evaluated the decisions that we would have taken had only the value of eGFR by the more used formulas (CKD-EPI Creatinine, MDRD, CKD-EPI Cystatine and CKD-EPI Creatinine-Cystatine).

### Statistical analysis

For the agreement analyses we used the statistical package AGP (Agreement Program) v.1.0 (Geiko, SP) available at: https://lfr.ecihucan.es/apps/?dir=agreement_installer. AGP is based on the R code originally developed by Lawrence Lin and YuYue. AGP was developed to simplify the use of the tool given in the R agreement package.

## Results

### Patients

A total of 315 patients were included, around 70% male, 38% had diabetic nephropathy and 18% nephroangiosclerosis (Table 1). Mean age was 66 ± 13 years. Most patients had hypertension and dyslipidemia and 40% had previous cardiovascular events. Nearly 50% were on an ACE inhibitor or ARB.

**Table 1:** Clinical characteristic of the patients included in the study.

N	N = 315
Age (y)	66.5 ± 13
Gender (male-%)	216 (69%)
Weight (kg)	81 ± 17
Height (m)	164 ± 19
BMI (kg/m^2^)	29 [25–33]
Renal disease (n%)	
Diabetic nephropaty	119 (38%)
Nephroangioesclerosis/renovascular	58 (18%)
Interstitial nephropaty	52 (16%)
Adquired policystic renal disease	17 (5%)
Primary glomerulonephritis	26 (8%)
Other	39 (12)
Comorbilities (n%)	
Diabetes mellitus	180 (57%)
Cardiovascular disease	127 (40%)
Dislipidemia	250 ( 79% )
Hypertension	299 (95%)
Hyperuricemia	200 (63%)
Smoking (n-%)	
Previous	144 (46%)
Active	35 (11%)
Medications (n-%)	
Insuline (%)	138 (44%)
Antyhipertensive	3 [2–3]
RAASi (%)	163 (52%)
Diuretics (%)	239 (76%)
Statins (%)	212 (67%)
Renal function	
Serum Creatinine (mg/dl)	3.2 ± 1.03
Serum Cistatin-C (mg/dl)	2.8 ± 1.08
24 h Cr Clearance (ml/min)	25.6 ± 10.3
Measured GFR (ml/min)	22.25 ± 8
mGFR < 15 ml/min	70 (22%)
mGFR 16–29 ml/min	207 (66%)
mGFR > 30 ml/min	37 (12%)
CKD-EPI (ml/min)	21.75 ± 8.44
MDRD (ml/min)	22 ± 8.3
CKD EPI CrCy	21.2 ± 7.5
CKD- Cy	22.33 ± 7.5
24 h urine proteinuria (mg/day)	1588 ± 2204
300 mg-3.5 g/24 h (n-%)	189 (60%)
> 3.5 g/24 h	43(14%)
Microalbuminuria (%)	76 (24%)
Albumin/Creatinine ratio (mg/g)	221 [3.7–806]
Laboratory test	
Haemoglobin (g/dL)	12.2 ± 1.4
HbA1c %	6.7 ± 1.5
Total cholesterol (mg/dl)	164 ± 41
Triglycerides (mg/dl )	155 ± 76
Potassium (mEq/dl)	5 ± 2.5
Phosphorus (mg/dl )	4 ± 0.8
PTHi (pg/ml)	200 ± 152
Bicarbonate (mmol/L)	26 ± 4.4

IQR interquartile range. RAASi: Renin angiotensin aldosterone system inhibitors; CKD- Cy: CKD EPI cystatin; CKD EPI CrCy: CKD EPI creatinine-cystatin; HbA1c: glycated haemoglobin.

### Renal function

Measured GFR was 22 ± 8 ml/min; 22% had mGFR < 15 ml/min, 27% 15–20 ml/min, 51% > 20 ml/min and about 12% > 30 ml/min (Table 1). Serum creatinine was 3.2 ± 1 mg/dl; mean eGFR was 22 ± 8 ml/min (CKD-EPI), 22 ± 8 ml/min (MDRD) or 26 ± 10 ml/min (24-h creatinine clearance). Cystatin C measurement was available in 266 patients (84%) and averaged 2.8 ± 1.08 mg/L. More than 70% of the patients had proteinuria: 1588 ± 2204 mg/24 h (Table 1).

### Agreement between eGFR and mGFR

#### Creatinine-based formulas

TDI averaged 70%, indicating that 90% of the estimations ranged from − 70% to + 70% of mGFR (Table 2, table S1 in Supplementary Information). No formula showed 90% of the estimations within ± 10% of mGFR. The majority of the equations had a CCC of about 0.6, reflecting a low level of precision and accuracy. No formula showed a CCC greater than 0.9. Finally, cp averaged 20% indicating that for each formula more than 80% of the estimations had an error greater than ± 10% (Table 2, table S1 in Supplementary Information).

**Table 2:** Agreement between mGFR y eGFR with different formulas.

	CCC	TDI	cp
Creatinine-based-formulas			
Cockcroft-Gault	0.59 (0.53)	71 (77)	22 (21)
aMDRD	0.63 (0.58)	62 (68)	25 (23)
MCQ	0.61 (0.55)	68 (74)	24 (22)
Rule-CKD	0.63 (0.57)	66 (72)	24 (23)
CKD-EPI-cr	0.63 (0.57)	64 (70)	25 (23)
Lund-Malmö (Rv)	0.63 (0.57)	58 (63)	26 (25)
FAS-cr	0.40 (0.35)	110 (119)	12 (11)
EKFC-cr	0.63 (0.57)	63 (68)	25 (24)
Cystatin-C-based			
Le Bricon	0.25 (0.22)	152 (162)	4 (3)
Rule-cy	0.66 (0.60)	57 (62)	27 (25)
Stevens-2	0.64 (0.58)	59 (64)	26 (25)
CKD-EPI-cy	0.68 (0.63)	55 (60)	28 (26)
FAS-cy	0.27 (0.23)	154 (164)	5 (4)
EKFC-cy	0.61 (0.55)	66 (72)	24 (23)
Creatinine-cystatin-C-based-formulas			
FAS-cr-cy	0.36 (0.31)	120 (128)	7 (6)
CKD-EPI-cr-cy	0.72 (0.67)	51 (56)	29 (27)
EKFC-crcy	0.70 (0.65)	52 (57)	29 (27)

CCC: concordance correlation coefficient; TDI: total deviation index; CP: coverage probability; Cy: cystatin; Cr: creatinine. In parentheses upper confidence interval (CI) .

#### Cystatin c–based formulas

TDI averaged 84%, meaning that 90% of the estimations of GFR showed an error ranging from − 84 to + 84% when compared with mGFR (Table 2, table S1 in Supplementary Information). No formula showed 90% of the estimations within bounds of error of ± 10% compared with the gold standard. The majority of the equations had a CCC of about 0.56, reflecting a low level of precision and accuracy. Finally, cp averaged 22 indicating that more than 78% of the estimations had an error range greater than ± 10%.

#### Creatinine- and cystatin c–based formulas

TDI averaged about 70% (Table 2, table S1 in Supplementary Information). No formula showed 90% of the estimations within ± 10% of mGFR. Most equations had a CCC of about 0.6 and cp averaged 24%.

P10 and p30 values: p10 average 20–30%, indicating that most of the cases have an error larger than 10% of mGFR. Also, p30 varied from 50 to 70%, indicating the half or 30% of the estimations had an error larger than 30% of mGFR (table S2 in Supplementary Information). Finally, the agreement analyses (TDI, CCC and cp) for GFR adjusted by BSA is shown in Supplementary Information (table S7 in Supplementary Information). The results are similar to those using unadjusted values.

No major difference were observed in the agreement analysis between patients with and without diabetes or in elderly subjects (Table S3 in Supplementary Information).

### Examples of the low agreement between eGFR and mGFR

Table 3 shows examples of the large discrepancies between eGFR and mGFR. In case 2 (mGFR 10 ml/min), eGFR ranged from 19 (MDRD) to 11.5 ml/min (CKD-EPI Cys). Among patients with higher mGFR, in case 9 (mGFR 25 ml/min) eGFR ranged from 13 (MDRD) to 19 ml/min (CKD-EPI Cys). Notably, the same formula in patients with identical mGFR (patients 6, 7, and 8, mGFR: 20 ml/min), could provide different eGFR values from 12 to around 25 ml/min (MDRD, CKD-EPI).

**Table 3:** Examples of overestimation or underestimation of measured GFR by estimated GFR. Patients grouped in cases with similar mGFR.

Cases	mGFR	MDRD	CKD-EPI cr	CKD-EPI Cys	CKD-EPI CrCys
1	8	23	22	11	15
2	10	19	19	11.5	14
3	10.5	14	15	16	14.3
4	15	19	18	18.5	20.5
5	15	13	12	13	12
6	20	12	12	20	15
7	20	24	25	32	27
8	20	24	25	32	27
9	25	13	14	19	11
10	25	31.5	34	32	31.5
11	31	29.5	29	25	26
12	31	48.5	49	35.5	26

**Figure 1:**
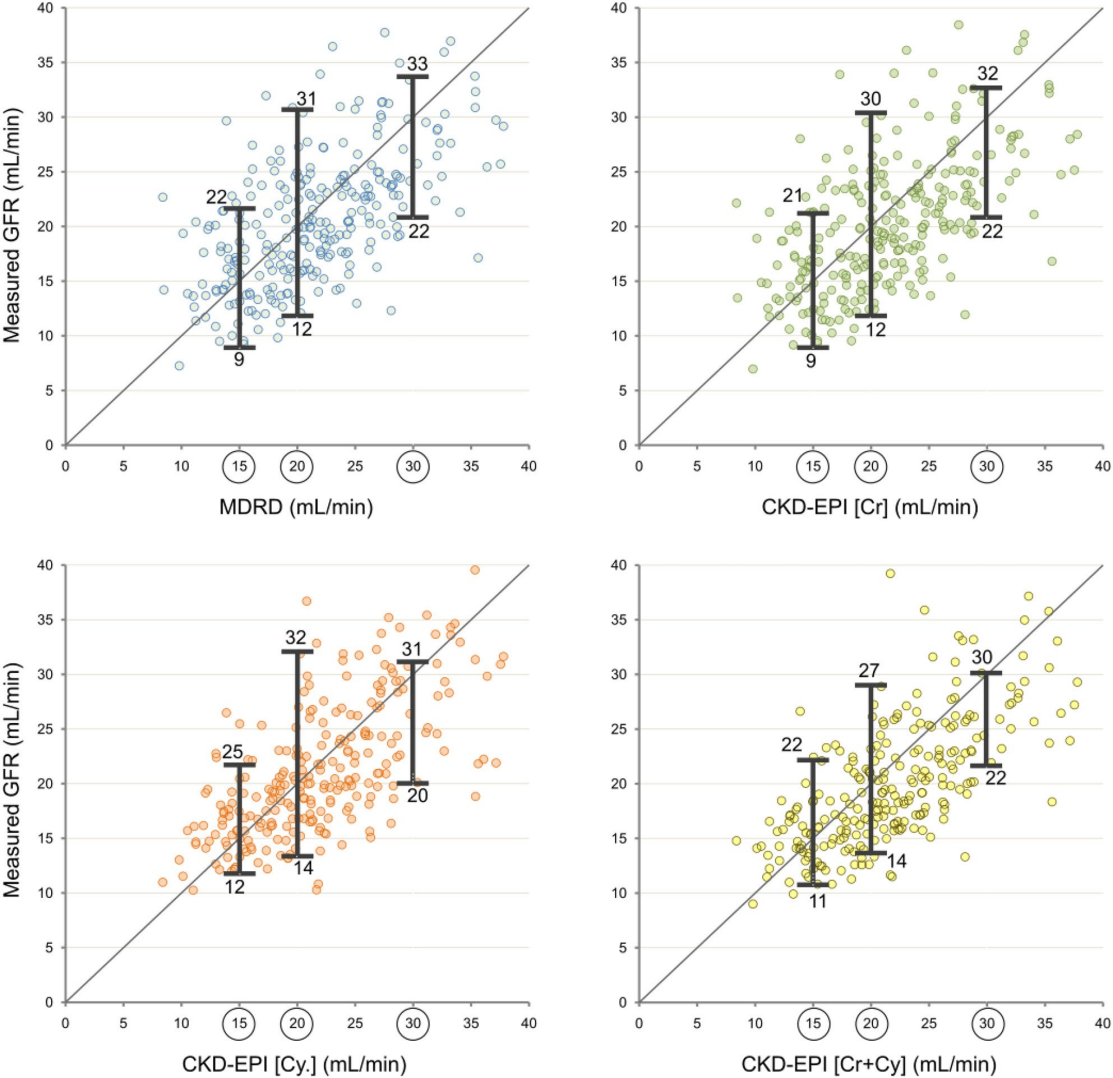
Variability between estimated GFR by different formulas: MDRD, CKD-EPI based on creatinine, cystatin- or both markers.

This large variability was observed in the whole population (Fig. 1). For values of mGFR such as 15, 20 and 30 ml/min, 4 different formulas provided eGFR values diverse from mGFR: for mGFR values of 20 ml/min, eGFR ranged from 12 to 30 ml/min (Fig. 1). Similar variability was found for the other values. Finally, in Fig. 1 it can be observed that some patients have levels of mGFR higher than 30 ml/min, reflecting the underestimation of eGFR in cases around this levels.

Finally, extreme differences between mGFR and eGFR were found. In nearly 20% patients with mGFR < 15 ml/min, eGFR was > 20 ml/min (MDRD, CKD-EPI-cr; Table S4 Supplementary Information). In contrast, in < 10% of the cases eGFR values were < 15 ml/min when actual mGFR was > 20 ml/min.

### Differences in clinical decision making

#### Clinical decisions based on mGFR

**Figure 2:**
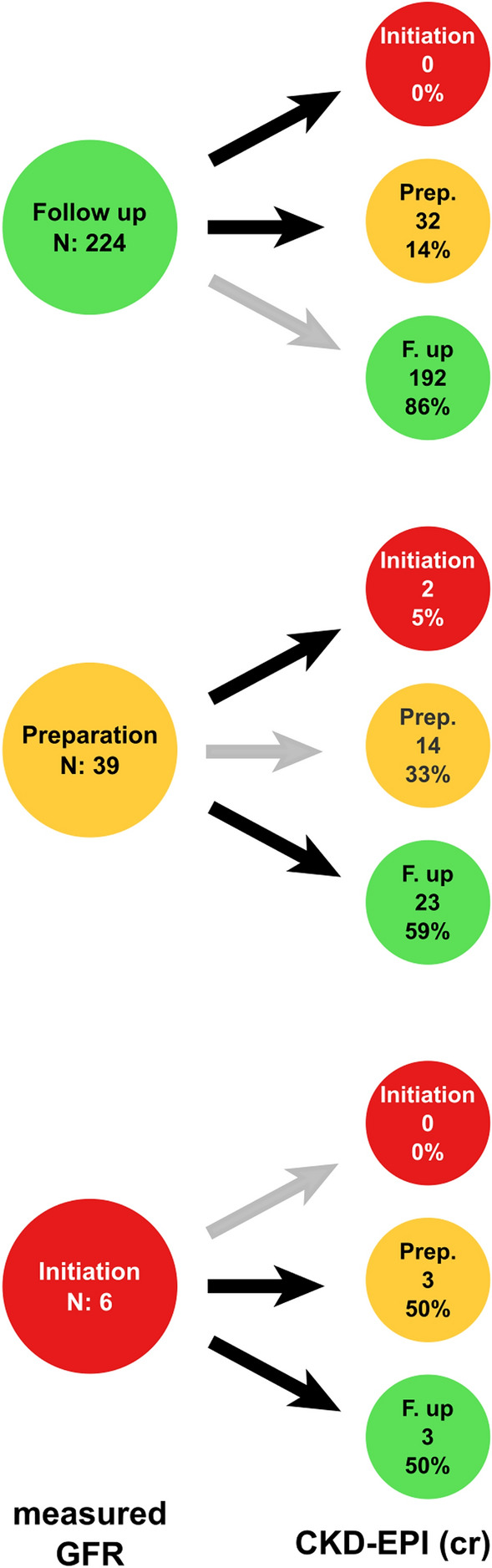
Differences in clinical decision based on mGFR or eGFR in patients with non-clinical indication of dialysis initiation.

For this analysis, eighteen subjects were excluded since dialysis was indicated shortly after the measurement of iohexol due to uremic symptoms or fluid overload, irrespective of the evaluation of GFR (group C). Thus, the analysis was performed in 269 patients. The mGFR levels at the time of starting dialysis of these cases is reported in Supplementary Information (Table S5, Supplementary Information). As expected most of these cases started dialysis with mGFR levels above 10 ml/min. Also, no data on follow-up after mGFR determination was available in 28 subjects. The remaining 269 patients were clinically stable without uremic symptoms and clinical decisions were only made according to the levels of mGFR. Among them, 224 (83%) were stable and continued with follow-up, 39 (14.4%) started the preparation for RRT and 6 (2.2%) started RRT (Fig. 2, Table S6 Supplementary Information.

#### Clinical decisions based on eGFR (CKD-EPI creatinine)

- *In those in whom regular follow up continued based on mGFR (n* = *224),* preparation for RRT would have been started in 32 patients (14%) due to eGFR underestimation of mGFR (Fig. 2—Table S6 Supplementary Information).
- *In those in whom the preparation for RRT started based on mGFR (n* = *39),* eGFR would have suggested to continue with follow-up in 23 (59%) due to eGFR overestimation of mGFR or to initiate dialysis early in 2 (5%) due to eGFR underestimation of mGFR (Fig. 2—Table S6 Supplementary Information).
- *In patients without symptoms who started RRT based only on low mGFR (n* = *6,2%),* eGFR would have suggest delaying this decision in all cases, due to eGFR overestimation of mGFR. Similar discrepancies were observed with other formulas (Table S6 Supplementary Information).

## Discussion

The error of formulas in patients in pre-dialysis case was large, frequent and random. The average error was about 60–70% of mGFR, making the estimation of mGFR by formulas an unreliable procedure. In clinical practice and decision-making perspective, this error may be particularly relevant, leading to incorrect evaluation of renal function and premature or late preparation RRT.

Formulas were unreliable in reflecting mGFR. Major over- or underestimation of mGFR were frequent, as indicated by TDI and CCC of 60–70% and 0.6 respectively. A TDI of 60–70% means that for a mGFR of 18 ml/min, eGFR may vary from 10 to 26 ml/min. These large variations affected about 90% of the cases. Acceptable errors i.e. ~ 10% were infrequent, as indicated by a cp of 20%. The error was, in some cases, random, since the estimations provided diverse (and even opposite) values in different patients with similar mGFRs. Finally, the error was observed with formulas using cystatin-c or creatinine, indicating that cystatin-c does not offer any advantage in GFR estimation.

The explanation of this issue is complex. Formulas are based on creatinine or cystatin-c levels, which do not reflect renal function properly^32^. Creatinine and cystatin-c may fluctuate depending on factors unrelated GFR. Creatinine is influenced by protein intake, muscle mass, renal tubular handling and extra renal clearance^14,33,34^. Thus, low-protein intake, sarcopenia or limb amputations influence creatinine independently of renal function^33^. Of relevance in predialysis patients, renal tubular cells can reabsorb and secrete creatinine. Creatinine secretion increases proportionally with CKD progression, leading to the overestimation of mGFR by formulas^33^. Finally, extra-renal clearance of creatinine, mainly by the gut, increases with CKD^14^. Cistatin-c can be influenced by different factors like hyper or hypothyroidism, smoking, gender, diabetes, metabolic syndrome and inflammatory status^35^. All these factors determine the known low correlation between these biomarkers and mGFR^36^ which could be as large as 200%^13^. Unfortunately, this error has not been solved by the creation of formulas, since the correlation between eGFR and mGFR is as wide as that of the single markers and mGFR^16,36,37^ (Fig. 1).

This error of eGFR may have relevant consequences in predialysis care. The main consequence of this error was the untimely preparation of patients for RTT. By the one hand, in patients with low GFR levels approaching the cut-off for dialysis, eGFR overestimation would have delayed general information on RRT, arterio-venous fistula creation or the start of pre-transplant protocols in almost 60% of the cases. Multidisciplinary education of patients is essential as it has been shown to improve overall survival and decrease healthcare costs in the first 6 months after dialysis initiation^38^ and its related with an increase of preemptive kidney transtplantation^39^. Unscheduled initiation of dialysis due to lack of functioning vascular access has been repeatedly associated with an increased risk of mortality in the first three months of dialysis due to infectious and cardiovascular events^40^. Also, in a small number of patients with GFR levels below the cut-off for starting dialysis (n = 6) and without uremic symptoms or fluid overload, RRT would have not been initiated based on eGFR, due to relevant overestimation of mGFR. By the other hand, an unnecessary fistula creation or intraperitoneal catheter insertion would have been performed, based on eGFR in 14% of the clinically stable patients. These anticipated clinical decisions may increase the severity of chronic comorbidities and decrease quality of life. Premature fistula placement may be associated with worse outcomes of heart disease due to an increase in left ventricular overload^48^. Also placement a peritoneal catheter have also risk associated to surgical risk and complications such as peritonitis^42^. Finally, in 5% of the cases in whom preparation for RTT was the option (2 of 39), dialysis could have been initiated based on false low levels of eGFR. This might have led to unnecessary insertion of central venous catheters, a fact that associated with worse survival outcomes.

Thus, trusting eGFR may lead to inadequate interventions with possible detrimental effects. Given the high-risk condition of this population, the possible error of eGFR in clinical decision must be considered in clinical care. We acknowledge that this sensitivity analysis must be tested in an ad-hoc designed investigation. Also, careful and attentive clinical monitoring is crucial in pre-dialysis care^43^. Finally, other indexes have been developed to predict the risk for disease progression^44^ and mortality in this population^45^. Measured GFR is, in any case, can be considered a useful tool to improve patient care in this high risk population.

Clinical guidelines recommend assessing eGFR in predialysis care^3–11^. However, the optimal timing to start RRT remains controversial, potentially due to the error inherent to assessing eGFR^46^. Thus, the GFR threshold to start dialysis has been established with formulas, based on studies that evaluated the risk and benefits of early or late dialysis initiation^4–26,28^. Some of them, found an increased mortality risk with early initiation of dialysis (eGFR > 10 ml/min)^8,47–49^. Also, many patients randomized to late initiation started dialysis with higher levels of eGFR, mostly due to uremic symptoms or fluid overload^46^. The low reliability of formulas in reflecting real GFR put those results into a different perspective. Formulas frequently overestimate or underestimate mGFR^36^. In those studies, patients with eGFR values near the cut-off to initiate dialysis may have had higher or lower levels of mGFR. Thus, its use in this high risk population should be questioned^46,48–50^. We are not claiming that creatinine and eGFR are not useful in day-to-day clinical practice. The eGFR is a good method to screen for chronic kidney disease, and to know when to refer a patient to nephrology. However, we are suggesting that in the era of precision medicine, the use of mGFR should be considered in specific conditions such predialysis patients, whenever possible.

The lack of precision and accuracy of eGFR has been shown in different settings, such as CKD^15^, polycystic kidney disease^16^, renal transplantation^17^, living kidney donors^51^, children with CKD^52^ etc. However, few studies analyzed the reliability of formulas in predialysis care. Evans et al.^37^ analysed the performance of several creatinine formulas with measured GFR by iohexol un patients with mGFR below 30 ml/min/1.73 m^2^. They observed that 35–45% of patients with CKD stage 4 and 5 the error of formulas was greater than +/− 30 of GFRm^44^.

Our study has limitations. Our cohort consisted mainly of Caucasians with a high representation of metabolic syndrome and obesity, so these results may not apply for other groups. Also, we need to consider that we took samples for iohexol determination up to 8 h after injection. A longer procedure i.e. up to 12 or even 24 hs would have been more accurate in patients with very low GFR. However, this is not a practical approach in clinical practice. Regarding the consequences of the error of eGFR in clinical decisions, particularly for the cases that started dialysis due to low GFR in the absence of uremia or fluid overload, the reduced number of cases ask for caution in the interpretation. Of note, the decisions that would have been made had we use eGFR instead of mGFR remains a simulation and shall be tested in ad hoc designed studies. On this regard, the study should be considered a hypothesis-generating study and this issue must be tested prospectively in ad-hoc designed, preferentially multicenter and international, investigations**.**

Further study in this area is needed, in particular the search of formulas with better accuracy and prediction in reflecting mGFR or the use of simplified gold standard techniques that facilitate the use of mGFR in the clinics, like the iohexol-DBS as used in our centre. Also, the impact of mGFR and mGFR changes over time and its impact in major outcomes in this population i.e. mortality and starting dialysis are worth investigating.

In conclusion, the known error of eGFR in patients with advanced renal disease is large, frequent and wide. This may have consequences in clinical decision in this group of patients.

### Supplementary Information

Supplementary Information.

## Data Availability

The data that support the findings of this study are available from Laboratory of Renal Function (ULL) but restrictions apply to the availability of these data, which were used under license for the current study, and so are not publicly available. Data are however available from the authors upon reasonable request from the correspondent author (esteban.l.porrini@gmail.com) and with permission of Laboratory of Renal Function (ULL).
