# Supplementary material for: The error of estimated GFR in predialysis care
Source: Sci Rep. 2024 Mar 3;14:5219. doi: 10.1038/s41598-024-55022-8 (PMC10909958; doi:10.1038/s41598-024-55022-8)
Supplement: Supplementary file 1 — Supplementary Information. [file 41598_2024_55022_MOESM1_ESM.pdf]

# THE ERROR OF ESTIMATED GFR IN PREDIALYSIS CARE

Authors: Escamilla-Cabrera Beatriz [1,8], Luis-Lima Sergio [2,4], Gallego-Valcarce E[3], Sánchez-Dorta Nuria[1], Negrín Mena Natalia[4], Díaz Martín Laura[4] , Cruz-Perera Coriolano[4] , Hernández-Valles Ana Montserrat[1], González-Rinne Federico[4] , Rodríguez-Gamboa María José[1], Estupiñán Torres Sara[1], Miquel Rodríguez Rosa[1], Cobo Caso Maria Angeles[1], Delgado-Mallén Patricia[1], Fernández Suárez Gema[3] , González Rinne Ana[1], Hernández Barroso Grimanesa[2] , González Delgado Alejandra[2] ,Torres Ramírez Armando[1], Jiménez Sosa Alejandro[5], Ortiz Alberto[6] , Gaspari Flavio[4] , Hernández-Marrero Domingo [1,7,8], and Porrini Estebani[4,7]

1.- Nephrology Department, Complejo Hospitalario Universitario de Canarias. La Laguna. Spain.

2.- Department of Laboratory Medicine. Complejo Hospitalario Universitario de Canarias. Tenerife. Spain

3.- Nephrology Department, Hospital Universitario Fundación Alcorcón . Madrid. Spain

4.-Laboratory of Renal Function (LFR), Complejo Hospitalario Universitario de Canarias. Faculty of Medicine, University of La Laguna, La Laguna. Spain.

5.- Research Unit, Hospital Universitario de Canarias. La Laguna. Spain.

6.- IIS-Fundación Jiménez Diaz, Department of Medicine, Faculty of Medicine, Universidad Autónoma de Madrid, Madrid. RICORS.

7.- Instituto de Tecnologías Biomédicas (ITB), Faculty of Medicine, University of La Laguna, La Laguna. Spain.

8. - Faculty of Medicine, University of La Laguna, La Laguna. Spain.

**Supplementary Appendix of the paper**

Table S1 . Agreement between mGFR y eGFR with different formulas .......................... 4

Table S2 p10 and p30 of the equations analysed in the study. ..................................... 6

Table S3: Agreement between DM and non DM, and > or < 65 years ........................... 7

Table S4: Percentage of cases that showed extreme overestimations or underestimation .......................................................................................................... 8

Table S5 mGFR of patients who started RRT due to uremic symptoms or fluid overload ................................................................................................................... 9

Table S6: Discordances in clinical attitude between mGFR and eGFR with different formulas excluding patients who initiated dialysis due to uremia or fluid overload .... 10

References of Lund-Malmö Equation ............................................................................ 11

Table S7: Agreement analysis for formulas adjusted by BSA ........................................13

| Creatinine-based-formulas | | | | | | | |
|---|---|---|---|---|---|---|---|
| | CCC | TDI | cp | | CCC | TDI | Cp |
| Effersøe | 0.59 (0.53) | 68 (74) | 23 (22) | Wright | 0.51 (0.46) | 79 (85) | 20 (18) |
| Edward-White | 0.36 (0.30) | 103 (112) | 16 (15) | MCQ | 0.61 (0.55) | 68 (74) | 24 (22) |
| Jelliffe-1 | 0.48 (0.41) | 107 (118) | 17 (16) | Sobh | 0.44 (0.38) | 92 (100) | 17 (16) |
| Mawer | 0.60 (0.55) | 70 (76) | 23 (21) | Rule-CKD | 0.63 (0.57) | 66 (72) | 24 (23) |
| Jelliffe-2 | 0.64 (0.59) | 60 (66) | 26 (24) | Virga | 0.57 (0.51) | 75 (81) | 21 (20) |
| Cockcroft-Gault | 0.59 (0.53) | 71 (77) | 22 (21) | CHUQ | 0.42 (0.35) | 93 (102) | 19 (17) |
| Björnsson | 0.53 (0.48) | 82 (88) | 19 (17) | CKD-EPI-cr | 0.63 (0.57) | 64 (70) | 25 (23) |
| Mogensen | 0.34 (0.28) | 147 (163) | 14 (13) | Lund-Malmö (LBM) | 0.57 (0.51) | 76 (82) | 21 (20) |
| Hull | 0.62 (0.56) | 69 (75) | 23 (22) | Lund-Malmö | 0.61 (0.55) | 61 (67) | 25 (24) |
| Gates | 0.64 (0.58) | 63 (69) | 25 (23) | Lund-1 | 0.56 (0.50) | 62 (67) | 25 (23) |
| Walser | 0.63 (0.57) | 67 (73) | 24 (22) | Lund-2 (LBM) | 0.49 (0.43) | 96 (104) | 16 (15) |
| Davis Chandler | 0.47 (0.39) | 75 (82) | 22 (20) | Lund-Malmö (Rv) | 0.63 (0.57) | 58 (63) | 26 (25) |
| Baracskay | 0.24 (0.17) | 105 (115) | 16 (15) | Lund-Malmö (RvLBM) | 0.61 (0.56) | 67 (72) | 23 (22) |
| Martin | 0.46 (0.41) | 101 (109) | 14 (12) | FAS-cr | 0.40 (0.35) | 110 | 12 (11) |
| aMDRD | 0.63 (0.58) | 62 (68) | 25 (23) | **EKFC-cr** | **0.63 (0.57)** | **63 (68)** | **25 (24)** |
| **Cystatin-C-based** | | | | | | | |
| | CCC | TDI | CP | | CCC | TDI | CP |
| Le Bricon | 0.25 (0.22) | 152 (162) | 4 (3) | Stevens-1 | 0.60 (0.54) | 64 (70) | 24 (22) |
| Tan | 0.55 (0.49) | 76 (83) | 20 (18) | Stevens-2 | 0.64 (0.58) | 59 (64) | 26 (25) |
| Hoek | 0.53 (0.47) | 77 (83) | 19 (18) | Tidman | 0.63 (0.58) | 69 (76) | 23 (22) |
| Larsson | 0.65 (0.60) | 57 (62) | 27 (25) | Grubb-2009 | 0.49 (0.42) | 94 (103) | 18 (17) |
| Perkins | 0.21 (0.18) | 200 (212) | 1 (1) | Hojs | 0.44 (0.39) | 98 (105) | 13 (12) |
| Orebro | 0.57 (0.52) | 95 (105) | 18 (17) | Grubb-2014 (CAPA) | 0.68 (0.62) | 58 (63) | 27 (25) |
| Grubb-2005 | 0.55 (0.49) | 88 (96) | 18 (17) | CKD-EPI-cy | 0.68 (0.63) | 55 (60) | 28 (26) |
| Rule-cy | 0.66 (0.60) | 57 (62) | 27 (25) | FAS-cy | 0.27 (0.23) | 154 | 5 (4) |
| MacIsaac | 0.44 (0.39) | 97 (105) | 13 (11) | **EKFC-cy** | **0.61 (0.55)** | **66 (72)** | **24 (23)** |
| Arnal-Dade | 0.69 (0.63) | 54 (59) | 28 (26) | MEAN (CAPA:RvLBM) | 0.72 (0.67) | 51 (55) | 30 (28) |
| Jonsson | 0.67 (0.61) | 58 (64) | 26 (25) | | | | |
| **Creatinine-cystatin-C-based-formulas** | | | | | | | |
| | CCC | TDI | CP | | CCC | TDI | CP |
| Ma | 0.67 (0.62) | 57 (62) | 27 (25) | FAS-cr-cy | 0.36 (0.31) | 120 | 7 (6) |

| | | | | | | | |
|---|---|---|---|---|---|---|---|
| Stevens | 0.73 (0.68) | 49 (53) | 31 (29) | **EKFC-crcy** | **0.70 (0.65)** | **52 (57)** | **29 (27)** |
| CKD-EPI-cr-cy | 0.72 (0.67) | 51 (56) | 29 (27) | | | | |

Table S1 . Agreement between mGFR y eGFR with different formulas. CCC: concordance correlation coefficient; TDI: total deviation index; CP: coverage probability; Cy: cystatin; Cr: creatinine. In parentheses upper confidence interval (CI) . Lund Malmö : Lund Malmö original equation. Lund-1 : Revised Lund Malmö formula without lean body mass. Lund -2 (LBM) : Revised Lund Malmö formula with lean body mass

| Creatinine-based-formulas | P10 | 95% CI | | P30 | 95% CI | |
|---|---|---|---|---|---|---|
| | | lower limit | Upper limit | | lower limit | Upper limit |
| **Effersøe** | **32** | 27 | 37 | **70** | 65 | 75 |
| **Edward-White** | **19** | 15 | 23 | **50** | 44 | 56 |
| **Jelliffe-1** | **23** | 18 | 28 | **58** | 52 | 64 |
| **Mawer** | **26** | 21 | 31 | **63** | 58 | 68 |
| **Jelliffe-2** | **30** | 25 | 35 | **75** | 70 | 80 |
| **Cockcroft-Gault** | **27** | 22 | 32 | **63** | 58 | 68 |
| **Björnsson** | **22** | 17 | 27 | **59** | 53 | 65 |
| **Mogensen** | **16** | 12 | 20 | **45** | 39 | 51 |
| **Hull** | **26** | 21 | 31 | **66** | 61 | 71 |
| **Gates** | **30** | 25 | 35 | **77** | 72 | 82 |
| **Walser** | **30** | 25 | 35 | **71** | 66 | 76 |
| **Davis Chandler** | **27** | 22 | 32 | **66** | 61 | 71 |
| **Baracskay** | **21** | 16 | 26 | **50** | 44 | 56 |
| **Martin** | **15** | 11 | 19 | **43** | 37 | 49 |
| **aMDRD** | **28** | 23 | 33 | **76** | 71 | 81 |
| **Wright** | **29** | 24 | 34 | **60** | 55 | 65 |
| **MCQ** | **23** | 18 | 28 | **71** | 66 | 76 |
| **Sobh** | **23** | 18 | 28 | **52** | 46 | 58 |
| **Rule-CKD** | **26** | 21 | 31 | **72** | 67 | 77 |
| **Virga** | **25** | 20 | 30 | **61** | 56 | 66 |
| **CHUQ** | **19** | 15 | 23 | **52** | 46 | 58 |
| **CKD-EPI-cr** | **26** | 21 | 31 | **73** | 68 | 78 |
| **Lund-Malmö (LBM)** | **27** | 22 | 32 | **65** | 60 | 70 |
| **Lund-Malmö** | **23** | 18 | 28 | **76** | 71 | 81 |
| **Lund-1** | **33** | 28 | 38 | **71** | 66 | 76 |
| **Lund-2 (LBM)** | **20** | 16 | 24 | **49** | 43 | 55 |
| **Lund-Malmö (Rv)** | **29** | 24 | 34 | **77** | 72 | 82 |
| **Lund-Malmö (RvLBM)** | **30** | 25 | 35 | **69** | 64 | 74 |
| **FAS-cr** | **13** | 9 | 17 | **40** | 35 | 45 |
| **EKFC_cr** | **28** | 23 | 33 | **75** | 70 | 80 |

| Cystatin-C-based | P10 | 95% CI | | P30 | 95% CI | |
|---|---|---|---|---|---|---|
| | | lower limit | Upper limit | | lower limit | Upper limit |
| Le Bricon | 4 | 2 | 6 | 14 | 10 | 18 |
| Tan | 20 | 15 | 25 | 55 | 49 | 61 |
| Hoek | 22 | 17 | 27 | 55 | 49 | 61 |
| Larsson | 33 | 28 | 38 | 74 | 69 | 79 |
| Perkins | 1 | 0 | 2 | 7 | 4 | 10 |
| Orebro | 21 | 16 | 26 | 59 | 53 | 65 |
| Grubb-2005 | 23 | 18 | 28 | 65 | 60 | 70 |
| Rule-cy | 31 | 26 | 36 | 80 | 75 | 85 |
| MacIsaac | 13 | 9 | 17 | 40 | 34 | 46 |
| Arnal-Dade | 36 | 30 | 42 | 81 | 76 | 86 |
| Jonsson | 33 | 28 | 38 | 80 | 75 | 85 |
| Stevens-1 | 28 | 23 | 33 | 67 | 62 | 72 |
| Stevens-2 | 33 | 28 | 38 | 72 | 67 | 77 |
| Tidman | 28 | 23 | 33 | 66 | 61 | 71 |
| Grubb-2009 | 24 | 19 | 29 | 62 | 56 | 68 |
| Hojs | 12 | 8 | 16 | 41 | 35 | 47 |
| Grubb-2014 (CAPA) | 36 | 30 | 42 | 77 | 72 | 82 |
| CKD-EPI-cy | 34 | 29 | 39 | 78 | 73 | 83 |
| FAS-cy | 4 | 2 | 6 | 17 | 13 | 21 |
| EKFC_cy | 32 | 27 | 37 | 70 | 65 | 75 |
| MEAN (CAPA:RvLBM) | 35 | 29 | 41 | 82 | 78 | 86 |
| **Creatinine-cystatin-C-based-formulas** | | | | | | |
| Ma | 32 | 27 | 37 | 73 | 68 | 78 |
| Stevens | 38 | 32 | 44 | 83 | 79 | 87 |
| CKD-EPI-cr-cy | 31 | 26 | 36 | 82 | 78 | 86 |
| FAS-cr-cy | 6 | 3 | 9 | 26 | 21 | 31 |
| EKFC_crcy | 34 | 29 | 39 | 79 | 74 | 84 |

Table S2 p10 and p30 of the equations analysed in the study.

| | | CCC | TDI | CP |
|---|---|---|---|---|
| **MDRD** | Non DM | 0.70 (0.61) | 53.68(61.48) | 0.28 (0.25) |
| | DM | 0.61 (0.52) | 65.23 (73.16) | 0.24 (0.22) |
| **CKD EPI** | Non DM | 0.711(0.62) | 53.19 (60.90) | 0.28 (0.25) |
| | DM | 0.60 (0.51) | 68.09 (76.42) | 0.23 (0.21) |
| **CKDEPI-Cy** | Non DM | 0.72 (0.64 ) | 47.87 (54.85) | 0.30 (0.27) |
| | DM | 0.66 (0.57) | 58.23 (65.55) | 0.26 (0.24) |
| **CKDEPI-CrCy** | Non DM | 0.76 (0.68) | 44.72 (51.15) | 0.32 (0.29) |
| | Non DM | 0.67 (0.59) | 57.11 (64.2) | 0.26 (0.24) |

| | | CCC | TDI | CP |
|---|---|---|---|---|
| **MDRD** | <65 years | 0.68(0.58) | 61.05(70.99) | 0.25(0.22) |
| | ≥65 years | 0.62(0.54) | 60.48(67.34) | 0.25 (0.23) |
| **CKD EPI** | <65 years | 0.68 (0.59) | 61.25(71.26) | 0.25 (0.22) |
| | ≥65 years | 0.62(0.54) | 62.76 (69.93) | 0.25 (0.23) |
| **CKDEPI-Cy** | <65 years | 0.64(0.53) | 58.82(68.53) | 0.26(0.23) |
| | ≥65 years | 0.711(0.64) | 51.25(57.22) | 0.29(0.26) |
| **CKDEPI-CrCy** | <65 years | 0.711(0.61) | 54.33 (63.11) | 0.27 (0.24) |
| | ≥65 years | 0.71 (0.64) | 50.81 (56.69) | 0.29 (0.27) |

Table S3: Agreement between DM and non DM, and > or < 65 years

| CREATINE-BASED EQUATIONS | | |
|---|---|---|
| | ***eGFR ≥ 20 aMDRD*** | ***eGFR ≥ 20 CKD-EPI-Cr*** |
| mGFR ≤15 vs eGFR ≥ 20 (N=60) | 13 (22%) | 10 (17%) |
| | ***eGFR ≤ 15 aMDRD*** | ***eGFR ≤ 15 CKD-EPI-cr*** |
| mGFR > 20 vs eGFR ≤ 15 (N=157) | 10 (6%) | 13 (8%) |
| **CYSTATIN-C BASED EQUATIONS** | | |
| | ***eGFR ≥ 20 CKD-EPI-cy*** | ***eGFR ≥ 20 CKD-EPI-CrCy*** |
| mGFR<15 vs eGFR>20 (n=53) | 24 (45%) | 4 (7.5%) |
| | ***eGFR ≤ 15 CKD-EPI-Cy*** | ***eGFR ≤ 15 CKD-EPI-CrCy*** |
| mGFR>20 vs eGFR<15 (n=148) | 10 (7 %) | 4 (3%) |

Table S4: Percentage of cases that showed extreme overestimations or underestimation by four formulas of estimation based on creatinine and/or cystatin-C, i.e. eGFR > 20 mL/min when mGFR was < 15 mL/min, and extreme underestimations i.e. eGFR < 15 mL/min when mGFR showed values > 20 ml/min.

| Case | mGFR (ml/min) | Cause for RRT | History Heart Failure |
|---|---|---|---|
| 1 | 14,6 | Fluid overload | Yes |
| 2 | 9,8 | Uremia | No |
| 3 | 15,3 | Fluid overload | No |
| 4 | 13,2 | Uremia | No |
| 5 | 14,3 | Fluid overload | Yes |
| 6 | 14,5 | Fluid overload | Yes |
| 7 | 18,3 | Uremia | No |
| 8 | 16,7 | Uremia | No |
| 9 | 28,1 | Fluid overload | No |
| 10 | 23,2 | Fluid overload/heart failure | Yes |
| 11 | 17,4 | Uremia | No |
| 12 | 8,4 | Uremia/metabolic acidosis | No |
| 13 | 22,3 | Uremia | No |
| 14 | 16,5 | Uremia | No |
| 15 | 29,1 | Fluid overload | Yes |
| 16 | 18,5 | Fluid overload/uremia | No |
| 17 | 13,8 | Fluid overload | Yes |
| 18 | 22,2 | Uremia | Yes |

Table S5 mGFR of patients who started RRT due to uremic symptoms or fluid overload

| Measured GFR N= 269 | | Estimated GFR | | | |
|---|---|---|---|---|---|
| | | MDRD | | CKD-EPI-creatinine | |
| **Clinical Follow-Up** | 224 (83) | *Follow-Up* | 198 (88) | *Follow-Up* | 192 (86) |
| | | *Preparation* | 14(6) | *Preparation* | 32(14) |
| | | *Initiation* | 12 (6) | *Initiation* | 0 |
| **Preparation for RRT** | 39 (15) | *Follow-Up* | 23 (59) | *Follow-Up* | 23 (59) |
| | | *Preparation* | 10 (26) | *Preparation* | 14 (36) |
| | | *Initiation* | 6 (15) | *Initiation* | 2 (5) |
| **Initiation of RRT** | 6 (2) | *Follow-Up* | 3 (50) | *Follow-Up* | 3 (50) |
| | | *Preparation* | 3 (50 ) | *Preparation* | 3(50) |
| | | *Initiation* | 0 | *Initiation* | 0 |
| **Measured GFR N=248** | | **CKD EPI Cr -Cy** | | **CKD- Cystatin-c** | |
| **Clinical Follow-Up** | 208 (84) | *Follow-Up* | 185 (89) | *Follow-Up* | 197 (95) |
| | | *Preparation* | 23 (11) | *Preparation* | 11 (5) |
| | | *Initiation* | -- | *Initiation* | -- |
| **Preparation for RRT** | 35 (14) | *Follow-Up* | 15 (43) | *Follow-Up* | 29 (83) |
| | | *Preparation* | 20 (57) | *Preparation* | 6 (17) |
| | | *Initiation* | -- | *Initiation* | -- |
| **Initiation of RRT** | 5 (2) | *Follow-Up* | 3 (60) | *Follow-Up* | 1 (20) |
| | | *Preparation* | 2 (40) | *Preparation* | 4 (80) |
| | | *Initiation* | -- | *Initiation* | -- |

Table S6: Discordances in clinical attitude between mGFR and eGFR with different formulas excluding patients who initiated dialysis due to uremia or fluid overload.

## References of Lund-Malmö Equation

- Björk J, Bäck E, Sterner G et al. Prediction of relative glomerular filtration rate in adults: New improved equations based on Swedish Caucasians and standardized plasma - creatinine assays, Scandinavian Journal of Clinical and Laboratory Investigation, 67:7,678-695

- Björk J, Grubb A, Sterner G et al. Revised equation for estimating glomerular filtration rate based on the Lund-Malmö Study cohort. Scand J Clin Lab Invest 2011; 71: 232–239.

| Creatinine-based-formulas | | | | | | | |
|---|---|---|---|---|---|---|---|
| | CCC | TDI | cp | | CCC | TDI | Cp |
| Effersøe | 0.56 (0.50) | 68 (74) | 23 (22) | Wright | 0.49 (0.43) | 79 (85) | 20 (18) |
| Edward-White | 0.39 (0.33) | 103 (115) | 16 (15) | MCQ | 0.60 (0.54) | 69 (75) | 24 (22) |
| Jelliffe-1 | 0.48 (0.42) | 110 (121) | 17 (16) | Sobh | 0.42 (0.36) | 93 (101) | 17 (16) |
| Mawer | 0.56 (0.50) | 70 (77) | 23 (21) | Rule-CKD | 0.60 (0.54) | 67 (73) | 24 (23) |
| Jelliffe-2 | 0.59 (0.52) | 61 (66) | 26 (24) | Virga | 0.53 (0.47) | 75 (82) | 21 (20) |
| Cockcroft-Gault | 0.53 (0.47) | 71 (77) | 22 (21) | CHUQ | 0.44 (0.37) | 95 (104) | 19 (17) |
| Björnsson | 0.48 (0.42) | 82 (89) | 19 (17) | CKD-EPI-cr | 0.61 (0.55) | 63 (71) | 25 (23) |
| Mogensen | 0.39 (033) | 151 (167) | 14 (13) | Lund-Malmö (LBM) | 0.49 (0.43) | 77 (84) | 21 (20) |
| Hull | 0.57 (0.50) | 70 (76) | 23 (22) | Lund-Malmö | 0.57 (0.51) | 62 (68) | 25 (24) |
| Gates | 0.61 (0.55) | 63 (69) | 25 (23) | Lund-1 | 0.50 (0.44) | 62 (67) | 25 (23) |
| Walser | 0.60 (0.54) | 67 (74) | 24 (22) | Lund-2 (LBM) | 0.40 (0.34) | 96 (104) | 16 (15) |
| Davis Chandler | 0.50 (0.43) | 75 (82) | 22 (20) | Lund-Malmö (Rv) | 0.60 (0.53) | 58 (63) | 26 (25) |
| Baracskay | 0.28 (0.21) | 105 (115) | 16 (15) | Lund-Malmö (RvLBM) | 0.55 (0.48) | 67 (72) | 23 (22) |
| Martin | 0.40 (0.34) | 102 (110) | 14 (12) | FAS-cr | 0.37 (0.31) | 111 (119) | 12 (11) |
| aMDRD | 0.61 (0.54) | 63 (69) | 25 (23) | **EKFC-cr** | **0.61 (0.55)** | **62 (67)** | **25 (24)** |
| **Cystatin-C-based** | | | | | | | |
| | CCC | TDI | CP | | CCC | TDI | CP |
| Le Bricon | 0.22 (0.19) | 152 (162) | 4 (3) | Stevens-1 | 0.58 (0.52) | 63 (68) | 24 (22) |
| Tan | 0.53 (0.47) | 76 (82) | 20 (18) | Stevens-2 | 0.61 (0.56) | 58 (63) | 26 (25) |
| Hoek | 0.51 (0.45) | 76 (82) | 19 (18) | Tidman | 0.63 (0.58) | 68 (74) | 23 (22) |
| Larsson | 0.64 (0.58) | 56 (61) | 27 (25) | Grubb-2009 | 0.66 (0.61) | 64 (70) | 18 (17) |
| Perkins | 0.19 (0.16) | 200 (212) | 1 (1) | Hojs | 0.42 (0.37) | 98 (105) | 13 (12) |
| Orebro | 0.58 (0.52) | 95 (105) | 18 (17) | Grubb-2014 (CAPA) | 0.68 (0.62) | 56 (61) | 27 (25) |
| Grubb-2005 | 0.55 (0.49) | 86 (94) | 18 (17) | CKD-EPI-cy | 0.68 (0.62) | 53 (58) | 28 (26) |
| Rule-cy | 0.65 (0.60) | 55 (60) | 27 (25) | FAS-cy | 0.25 (0.21) | 154 (164) | 4 (3) |
| MacIsaac | 0.41 (0.36) | 97 (105) | 13 (11) | **EKFC-cy** | **0.57 (0.50)** | **65 (71)** | **24 (23)** |
| Arnal-Dade | 0.67 (0.62) | 53 (57) | 28 (26) | MEAN (CAPA:RvLBM) | 0.69 (0.64) | 50 (54) | 30 (28) |
| Jonsson | 0.67 (0.61) | 57 (62) | 27 (25) | | | | |
| **Creatinine-cystatin-C-based-formulas** | | | | | | | |
| | CCC | TDI | CP | | CCC | TDI | CP |
| Ma | 0.66 (0.60) | 56 (61) | 27 (25) | FAS-cr-cy | 0.33 (0.29) | 120 (128) | 7 (6) |

| | | | | | | | |
|---|---|---|---|---|---|---|---|
| Stevens | 0.71 (0.66) | 48 (52) | 31 (29) | **EKFC-crcy** | **0.67 (0.61)** | **52 (57)** | **29 (27)** |
| CKD-EPI-cr-cy | 0.71 (0.65) | 51 (55) | 29 (27) | | | | |

Table S7: Agreement analysis for formulas adjusted by BSA
